# Towards a nationwide implementation of a standardized nutrition and dietetics terminology in clinical practice: a pre-implementation focus group study including a pretest and using the consolidated framework for implementation research

**DOI:** 10.1186/s12913-019-4600-5

**Published:** 2019-11-29

**Authors:** Gabriele Gäbler, Michaela Coenen, Katrin Fohringer, Michael Trauner, Tanja A. Stamm

**Affiliations:** 1 Section for Outcomes Research, Center for Medical Statistics, Informatics, and Intelligent Systems, Medical University of Vienna, Spitalgasse 23, 1090 Vienna, Austria; 2 LMU Munich, Department of Medical Information Processing, Biometry and Epidemiology (IBE), Chair of Public Health und Health Services Research, Marchioninistr. 17, 81377 Munich, Germany; 3 Pettenkofer School of Public Health, Munich, Germany; 4 Department of Medicine III, Division of Gastroenterology and Hepatology, Medical Nutrition Therapy and Dietetics, Vienna General Hospital, Währinger Gürtel 18-20, 1090 Vienna, Austria; 5 Department of Medicine III, Division of Gastroenterology and Hepatology, Medical University of Vienna, Währinger Gürtel 18-20, 1090 Vienna, Austria

**Keywords:** Nutrition therapy, Dietetics, Documentation, International classification of functioning, disability and health, Focus groups, Diffusion of innovation, Implementations science, Consolidated framework of implementation research (CFIR), Barriers and facilitators, Theories

## Abstract

**Background & Aims:**

In order to assure high quality of nutrition and dietetic care as well as research, the implementation of a standardized terminology, such as the World Health Organization (WHO) International Classification of Functioning, Disability and Health for Dietetics (ICF-Dietetics) is indispensable. The aim of this study was to explore the clinical practicability and applicability of the ICF-Dietetics in the field of nutrition and dietetic practice prior to the implementation in order to develop criteria (points to consider) for a targeted implementation strategy.

**Methods:**

A focus group study including a pretest of the ICF-Dietetics was conducted. Subsequently, facilitators and barriers for a nationwide implementation of the ICF-Dietetics in clinical nutrition and dietetic practice were identified and linked to interventions (combining theory-based and group-based approach) using the Consolidated Framework of Implementation Research (CFIR) to organize and represent data and summarized in a logic model.

**Results:**

In the pretest 55 clinical documentations which consisted of 248 different ICF-Dietetics categories were received. In four focus groups with 22 health professionals, 66 relevant higher-level themes and implementation strategy criteria (points to consider) were identified. These themes referred to all five domains of the CFIR, namely intervention characteristics, inner setting, outer setting, characteristics of individuals and implementation process and contained important barriers and facilitators that were linked to six implementation objectives as well as six context requirements and five main actors.

**Conclusions:**

This study provides facilitators and barriers to be addressed when implementing the ICF-Dietetics in clinical practice and shows potential interventions based on this analysis. A nationwide implementation was mainly seen as a great advantage for enhancing quality and continuity of care and for providing comparable data. However, it requires further refinements and a multifaceted implementation strategy where the engagement of leadership of institutions plays a crucial role. These results have provided a foundation for a targeted implementation strategy to increase the success, reproducibility and comparability.

## Background

Health conditions related to nutrition, such as diabetes, obesity, oncology (various kinds of cancer), gastrointestinal diseases or surgical interventions require medical nutrition therapy [1–3]. Dietitians and nutritional physicians are responsible for medical nutrition therapy. Dietitians are non-physician health professionals who deliver their interventions either to individual patients or groups of patients by applying the nutrition care process (NCP) and dietetic care process (DCP), respectively, designed to improve and standardize the consistency and quality of dietetics care [1, 4–7]. In order to facilitate high quality and consistency of care to all European citizens including cross-border interfaces, dietitians need to consistently apply the NCP or DCP with a standardized terminology all over Europe [1, 8, 9]. While some countries have already implemented a standardized terminology, several are still lacking one.

Two standardized nutrition and dietetics terminologies exist in nutrition care worldwide: (1) the well-established Nutrition Care Process Terminology (NCPT) developed by the American Academy of Nutrition and Dietetics which is exclusively a nutrition and dietetics terminology and (2) the International Classification Functioning, Disability and Health (ICF) for Dietetics (ICF-Dietetics) a derived classification of the World Health Organizations (WHO) tailored to the needs in dietetics care. The ICF-Dietetics was accepted by the Dutch WHO collaboration center of the WHO Family of International Classifications (WHO-FIC) Network. It was found suitable to describe concepts for the assessment, dietetics diagnoses, intervention goal setting and evaluation [10, 11]. The ICF-Dietetics enlarges the ICF by adding 900 specific categories addressing nutrition and dietetics related issues and could be considered valuable regarding a potential usage in multidisciplinary team care [11]. While the applicability of the NCPT [12–15] and its implementation [13, 16–21] as well as the ICF without its extension specific to dietetics care has been evaluated [22–31], there is a lack of data on the practicability and applicability of the ICF-Dietetics in clinical dietetic practice.

In order to successfully implement complex interventions, an elaborated strategy designed to address identified barriers is recommended [32]. In that regard, Leeman et al. [33] provided a structured overview of strategies to facilitate reporting of implementation research findings and alignment to relevant theories. To use such theoretical approach and framework when conceptualizing an implementation strategy is widely endorsed [34–36], while still opposed by some scholars [37]. Nevertheless, an advantage of such a framework is that it allows researchers to use a common language to better synthesize and compare findings across settings and interventions [34, 38, 39]. In the literature, a large number of frameworks, models and theories for transferring research into practice exist which are useful for effective implementation [34, 40]. In order to select the most appropriate theoretical approach, useful information is provided in literature [40]. For example, the Consolidated Framework for Implementation Research (CFIR) [38] is based on 19 different implementation models and frameworks (e.g. the PARiHS Framework) and provides detailed and clearly defined constructs and domains for the implementation process and its context. To date, several studies have used the CFIR to identify barriers and facilitators (determinants of healthcare practice that affect the intervention) in pre-, during or post-implementation of clinical innovations [39, 41–45]. Only few studies so far, e.g. Robins et al. [44], used the CFIR prior to implementation of the clinical innovation to identify potential barriers and facilitators, although, this is recommended [38, 46, 47]. In that regard, evaluation in the planning phase allows for fine-tuning of the new concept before resources are spent on an insufficient implementation [42, 46]. Identifying barriers and facilitators of healthcare practice is one phase in the complex and multifaceted process of getting new innovations into practice. This phase is followed by linking specific tailored interventions to these determinants before applying and evaluating the intervention in practice [48–50]. The effects of tailored interventions are non-exhaustively examined [51–55]. To this end, precise descriptions to get comparable data across interventions are needed [56, 57].

The aim of this pre-implementation study was to explore clinical practicability and acceptability of the ICF-Dietetics prior to its implementation in order to inform a targeted implementation strategy. The specific objectives were (a) to develop an application concept and educational program to incorporate the ICF-Dietetics in clinical dietetic practice, (b) to pretest the ICF-Dietetics in clinical practice by trained target users (dietitians), (c) to explore the acceptability of the ICF-Dietetics and its application concept with respect to a prospective nationwide implementation by means of focus groups, (d) to identify barriers and facilitators for the implementation according to CFIR constructs, and (e) to link interventions, theories and responsibilities to these identified barriers and facilitators in order to develop a logic model for the implementation of the ICF-Dietetics in clinical practice.

## Methods

### Study design

**Fig. 1:**
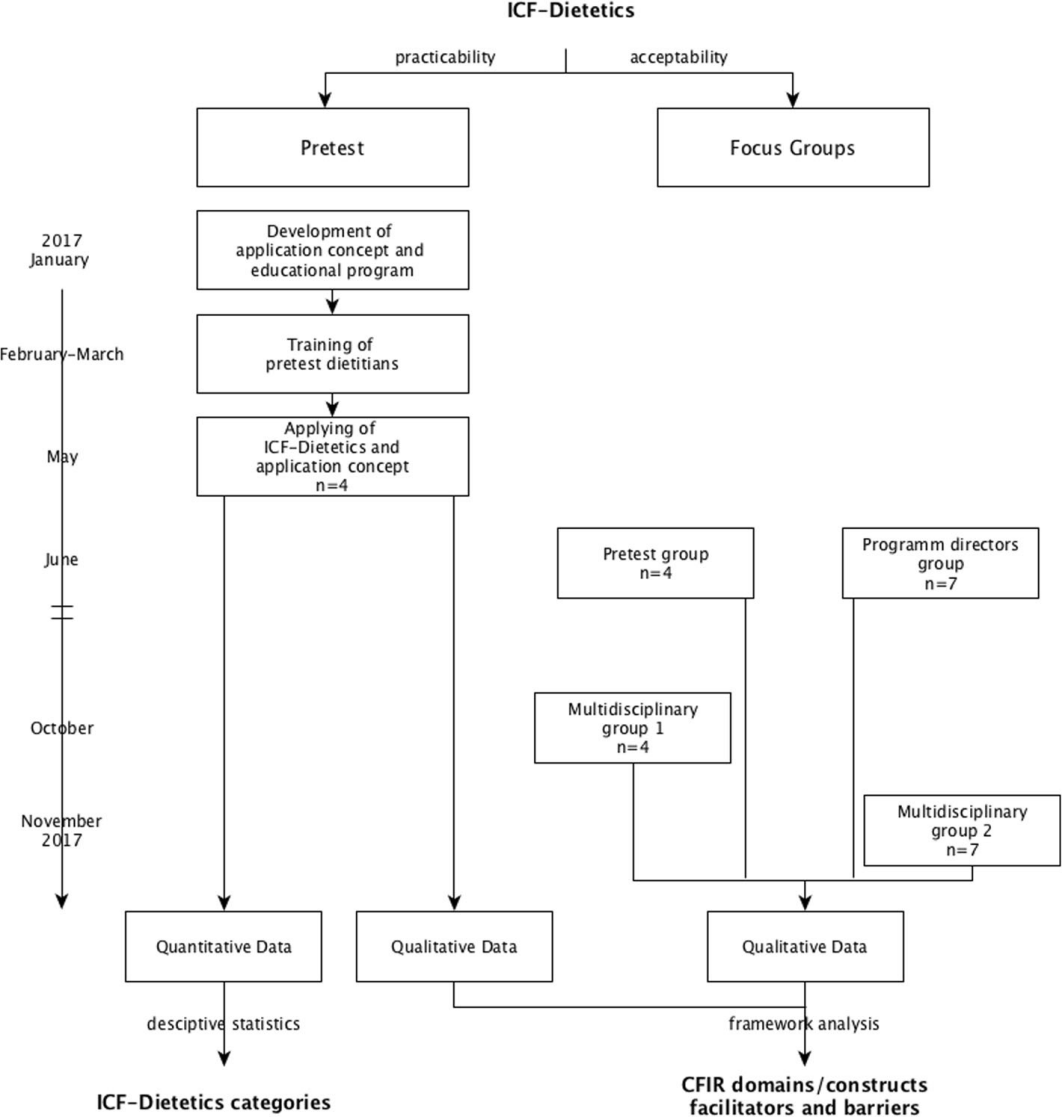
Study flow chart with timeline

A focus group study (extreme case sampling) was conducted including a pretest of the ICF-Dietetics applied by dietitians (self-selection sampling) after having completed an educational program and using the Consolidated Framework of Implementation Research (CFIR) to organize and represent data. A flow chart of the study process with a timeline is depicted in Fig. 1.

### Development of the application concept and the educational program

The application concept aiming to incorporate the ICF-Dietetics in clinical dietetic practice was developed for this study (GG) and builds on a previous study regarding clinical dietetic care documentation analysis [10] and the ICF-based documentation tools of the established multidisciplinary rehabilitation management approach, the ICF-based Rehab-Cycle [24, 25]. The tools (see Additional file 1), such as, the ICF-Dietetics Assessment Sheet and the ICF-Dietetics Categorical and Goal Profile were adapted and designed to apply within the NCP/DCP [10]. The application concept and the educational program for training of pretest participants, were established (GG) in January 2017 and validated by two academic dietitians by means of cognitive debriefing using face-to-face interview. The educational program consisted of two parts, firstly of an introduction regarding the biopsychosocial model of the ICF (duration 1–2 h) and secondly of a training focusing on the application of the ICF-Dietetics in clinical practice (duration 4–6 h). The training was held in terms of interactive presentation and practical exercises by examples.

### Determine practicability of the ICF-dietetics by means of a pretest

For this pretest, a large university hospital was chosen in which 22 dietitians (17.75 40-h full-time equivalents) are employed. Self-selection sampling was used, meaning that all 22 dietitians of this hospital were invited to the first introduction part. Five of them decided to participate in the educational training and four to apply the ICF-Dietetics for 3 weeks in May 2017 (after having applied the concept in 2–3 training patients). All four of them fulfilled the eligible criteria, such as, having clinical experience of at least 1 year and currently working in one of the most common dietetic medical areas, like gastroenterology, metabolic diseases, oncology, surgery, nephrology, intensive care, geriatrics and pediatrics. Participants of the study were provided the ICF-Dietetics in terms of a web-based version with a search function and had to achieve two aims (1) to apply the Dietetics Assessment Sheet and the ICF-Dietetics Categorical and Goal Profile (see Additional file 1) for approximately ten consultations of different medical areas and (2) to make notes about their experiences during the application phase. The qualitative notes were analyzed in a later stage of the project together with the focus group data. Descriptive statistics (absolute and relative frequencies) were used to depict the number of used ICF-Dietetics categories by medical areas. For better comparison with other studies, ICF-Dietetics categories were presented at the second level of the ICF classification. The framework of the ICF and the ICF-Dietetics, respectively, including the levels of the classification is described elsewhere [10, 11, 58].

### Determine acceptability of the ICF-dietetics implementation by means of focus groups

#### Focus group participants

Participating experts were recruited with a purposive extreme case sampling focusing on participants with unique or special characteristics to get insight into the topic at great depth. One group was conducted with dietitians who had taken part in the pretest of the ICF-Dietetics. Another group included all program directors of universities for dietetics of the country, and two further groups consisted of interdisciplinary teams of physicians and health professionals who had been applying the ICF in their clinical practice. All focus groups were natural groups with established working relationships. The participants received an introduction to the ICF-Dietetics, their underlying biopsychosocial model and the concept how to apply it in clinical dietetic practice (15–90 min). The pretest and program directors’ groups received a more detailed introduction compared to the multidisciplinary groups. Thus, all participants of the multidisciplinary groups were familiar with the ICF and its clinical application, and therefore were only given an introduction into ICF-Dietetics and its application. Health professionals of the multidisciplinary groups were eligible if they had clinical experience of at least 1 year and had been actually working with the ICF in clinical practice. According to previous studies [59, 60], the focus group size was set at a maximum of seven people to enable interactions and different opinions.

#### Data collection

To guide the focus group sessions, semi-structured, open-ended questions were developed (GG) and validated by a second experienced qualitative researcher (TS). All focus groups were moderated by a trained and experienced researcher (GG) together with one assistant responsible for observing the group and taking field notes during the discussion. In all groups, the same interview schedule (see Additional file 2) was used, however, allowing flexibility. The discussion started after a brief introduction of the participants (as an “icebreaker”). The focus groups were held at the institutions of the study participants and lasted between 45 and 60 min (without the introduction of the ICF-Dietetics). Attention was paid to a comfortable undisturbed conversation climate. Each focus group was audio-recorded and transcribed verbatim by one researcher (KF) using defined transcript rules.

#### Data analyses

For this study, framework analysis (a kind of thematic analysis) was used [61, 62]. This method provides clear and transparent steps and produces a highly-structured output. As described by Keith et al. [39], two frameworks (coding templates) were applied, firstly, a framework based on intervention-specific codes, secondly, the codebook of the CFIR [63]. This framework was chosen since it offers a guide for evaluating the entire implementation process in combination with other process framework e.g. the RE-AIM framework (Reach, Effectiveness, Adoption, Implementation, and Maintenance) [38, 64, 65]. The CFIR provides a pragmatic meta-theoretical structure with a high level of operationalization across constructs and domains [40], thus enables comparison of ratings across institutions [45] and studies. Moreover, it is widely used among health care studies [42, 66] for evaluations in terms of guiding data collection, analysis and reporting [39, 41, 45, 67, 68]. After familiarization with the verbatim transcripts (including the qualitative notes of the pretest), the text was divided into segments. Segments are meaningful parts of the text that belong together and were assigned with a conceptual label (code). The first coding framework emerged from the analysis of the first focus group transcript. Each code was discussed by two researchers regarding its meaning (GG and TS). Subsequently, this thematic framework (based on codes incorporating interview background and identified theme) was applied to all documents. However, new codes were created during this analyzing process when needed. In the next step, the codes were reduced, summarized and grouped around similar and interrelated themes. While codes were closely and explicitly linked to the raw data (original text), the process of assigning higher-level themes started the abstraction of the data. This abstraction was further developed by grouping codes and corresponding higher-level themes according to the CFIR domains, constructs and sub-constructs in a spreadsheet, generating a matrix. Additionally, this matrix depicted illustrative quotations and references to whether it was mentioned as a barrier (aspects everything that restrains or hinders implementation) or a facilitator (aspects everything that makes implementation easier or enables it). Table 1 illustrates the code reduction and abstraction process in terms of an example. The assignments (barrier or facilitator) were done with regard to explicit statements of focus group participants or interpretation of the context by the consensus of all researchers (GG, MC, KF, MT, TS). Data analysis was performed using ATLAS.ti Version 8.1.3, Scientific Software Development GmbH, Berlin.

**Table 1:** Example of framework analysis in terms of code reduction and charting process (matrix)

Code reduction process		Charting data into a matrix				
First Thematic Framework	Reduced Thematic Framework	CFIR Domain (construct)	Higher-level-theme	Quotation	F^a^	B^b^
Advantages/Strengths/Opportunities	Advantages/Strengths/Opportunities					
ICF-Dietetics and concept: Interdisciplinarity/ Multidisciplinarity	no change	Innovation Characteristics (Relative Advantage)	Interdisciplinarity/Multidisciplinarity	*“[...] I think that is very important, that we can work interdisciplinary and with other professional groups.”* [FG2_director_26y experience]	F	
Actual use of the ICF: Interdisciplinary/ Multidisciplinary collaboration	Actual use of the ICF: Interdisciplinarity/ Multidisciplinarity	Inner Setting (Networks & Communications)	Interdisciplinarity/Multidisciplinarity	*“So, we have an IT-technical network, where all professional group see, if* you [to the speech therapist] *changes something.”* [FG3_dietitian_18y experience]	F	
Disadvantages/ Weaknesses/Risk	Disadvantages/ Weaknesses/Risk					
ICF-Dietetics and Concept: Hindering interdisciplinarity	ICF-Dietetics and concept: Interdisciplinarity/ Multidisciplinarity	Inner Setting (Networks & Communications)	Interdisciplinarity/Multidisciplinarity	*“If the chef does not know what that is [...] it would not be anything good if it would be a sole dietitian thing.”* [FG3_physician_30y experience]		B
Prerequisites for Implementation	Prerequisites for Implementation					
Pay attention to other professional groups	Interdisciplinarity/ Multidisciplinarity	Inner Setting (Networks & Communications)	Interdisciplinarity/Multidisciplinarity	*“[...] the problem of a profession-specific language is [...] when it is not permeable to other professions.”* [FG3_physician_30y experience]		B
Effort of persuasion on multiprofessional approach	Persuading/Motivation	Characteristics of Individuals (Knowledge & Beliefs about the Innovation)	Interdisciplinarity/Multidisciplinarity	*“[...] and that’s why I just believe it takes persuasion that dietetics is part of the multiprofessional team.* [FG2_director_27y experience]	F	

^a^F = Facilitator (anything makes implementation easier or enables it)

^b^B = Barrier (anything restrains or hinders implementation

### Linking interventions, theories and responsibilities

The linking of interventions and responsibilities to identified barriers and facilitators was performed in two parts: firstly, a theory-based linking exercise of focus group results and literature [48, 49, 52, 53, 69–71] done by the first author (GG) and secondly, by an explorative group-based approach by different academic and/or clinical experts and authors of this study (GG, MC, KF, MT, TS). Subsequently, by merging the results according to implementation objectives and its context requirements with respect to its responsibilities, a logic model for the implementation strategy was developed.

## Results

### Descriptive results of the pretest

From the pretest, 55 documents (ICF-Dietetics Categorical and Goal Profiles) with a total number of 485 ICF-Dietetics categories (including duplicates) were received corresponding to 248 different ICF-Dietetics categories. The detailed descriptive statistics stratified by medical areas is shown in Table 2. The list of the actual second level ICF categories is provided in Additional file 3.

**Table 2:** Descriptive statistic of applied ICF-Dietetics categories in respect of medical areas

	Total	Diabetes and Metabolism	Gastroenterology	Surgery	Oncology	Others ^a^
Frequency of documents (n)	55	17	7	15	9	7
Frequency of extracted different ICF categories (n)	248 ^d^	102	45	102	54	48
Percentage (%) of total concepts *n* = 248		41	18	41	22	19
Frequency of second-level ICF categories (n)	75 ^d^	38	24	41	28	24
Percentage (%) of total *n* = 75		51	32	55	37	32
Body Functions (n)	32 ^d^	14	15	20	18	12
Body Structures (n)	5 ^d^	2	2	3	3	1
Activities (n) ^b^	15 ^d^	10	1	5	3	3
Participation (n) ^b^	6 ^d^	1	2	4	3	0
Environmental Factors (n)	10 ^d^	8	4	4	1	3
Personal Factors (n) ^c^	7 ^d^	3	0	5	0	5

^a^Other medical areas included nephrology, pediatrics, neurology

^b^In contrast to the original ICF where “Activities and Participation” begins with (d), the ICF-Dietetics differentiates between “Activities (a)” and “Participation (p)” as it is also given as an alternative option by World Health Organization [58]

^c^ICF-Dietetics provides a first draft of codes covering “Personal Factors”

^d^A concept could be used in different medical areas, thus, n is not the sum of them

### Focus group dynamics

In total, four focus groups with 22 participants were conducted. Focus groups characteristics are depicted in Table 3. All participants mostly supported the use of a standardized terminology in general and the use of the ICF-Dietetics in particular. The main topic of discussion in the first pretest focus group was characterized by the positive attitude towards a common terminology on the one hand and by the difficulties in the practical application of the ICF-Dietetics on the other hand. The second focus group with the program directors was dominated by an ambitious discussion on how to implement the ICF-Dietetics nationwide in clinical practice and in education. The multidisciplinary groups (third and fourth focus group) reflected the actual clinical practice of applying the ICF in the institutions in which the participants were employed. Consequently, specific practical aspects for implementing the ICF in a daily routine within an interdisciplinary setting were discussed.

**Table 3:** Focus group characteristics

Focus group	Number of participants	Health professions	Gender		Years of work experience					Highest degree of education		
			Female	Male	Median	Mean	SD	Min	Max	Bachelor	Master	Medical specialist
1	4	Dietitians (pre-test)	3	1	2	4.2	4.5	2	11	2	2	–
2	7	Dietitians (program directors)	6	1	27	27.1	9.4	14	40	–	7	–
3	4	Physician, Dietitian, Nurse, Speech and Language therapist	3	1	12	14.8	11.8	4	30	2	1	1
4	7	Physician, Dietitian, Nurse, Speech-, Physiotherapist, Linguist	5	2	25	23.4	6.3	15	31	4	1	2
Total	22		17	5	19.5	19.5	11.5	2	40	8	11	3

### Identified barriers and facilitators for a nationwide implementation of the ICF-dietetics listed by CFIR domains and constructs

**Fig. 2:**
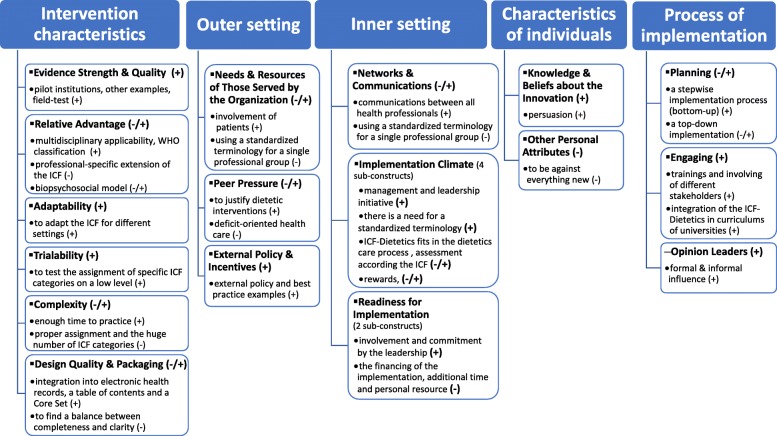
Summary of facilitators and barriers according to CFIR domains and constructs (+ refers to facilitators, and – refers to barriers)

Initially, 163 codes were identified in the first thematic framework, that were reduced to 133 codes representing 66 higher-level themes, which refer to all five CFIR domains and to 21 of total 36 CFIR constructs. While in 19 constructs facilitators for a prospective implementation were identified, barriers were allocated to 11 constructs. Identified barriers and facilitators according to each domain and subordinated constructs are reported below. A summary of these findings depicts Fig. 2. Frequencies how often the topic was discussed in terms of domains/constructs and higher-level themes, as well as derived criteria for a nationwide implementation are outlined in Additional file 4.

### CFIR domain intervention characteristics

#### Evidence Strength & Quality

Stakeholders’ perceptions of the quality and validity of evidence supporting the belief that the innovation will have desired outcomes [38, 63]. Focus group participants mentioned that it would be necessary to plan and start the implementation strategy with pilot institutions and to look at examples of other health professions or other countries. Additionally, the performed field-testing during the validation process of the ICF were discussed. (**Facilitator).**

#### Relative advantage

Stakeholders’ perception of the advantage of implementing the innovation versus an alternative solution [38, 63]. Besides the implementation of the NCPT, two possibilities were brought up, namely, no standardized terminology at all or developing a completely new dietetics language from the very beginning, however, both not seen as an alternative. As main advantage of implementing the ICF-Dietetics compared to the NCPT the multidisciplinary/interdisciplinary applicability was most often mentioned. A further advantage and a facilitator for the implementation of the ICF-Dietetics was mentioned, is the fact that the ICF is a reference classification of the WHO and has no license fee. (**Facilitator)**

*"But in principle I think, it is certainly clever not to develop a new language [...], but to apply the ICF, which is very widespread in the European area."* [FG4_physician_31y experience]

Additionally, many other general advantages (e.g. comprehensibility, comparability, standardization, professionalization etc.) compared to having no standardized terminology were discussed. For example, ICF-Dietetics with the biopsychosocial approach will facilitate structured and transparent care to gain additional information that otherwise may be missed. Moreover, specific advantages for patients in terms of continuity of care, patient centered care and better understanding of goals were pointed out. (**Facilitator).**

Not many general disadvantages of the ICF were mentioned. In one group the use of a professional-specific extension of the ICF was seen as great barrier for multidisciplinary team work. Additionally, while the biopsychosocial model was partly seen as supportive, it was emphasized by one expert that the usage of this model poses a crucial barrier. **(Barrier).**

*“This scheme with the biopsychosocial model is a major barrier in the dissemination of the ICF. Simply because body functions, activities and participation are placed on the same level. But body functions and activities and participation do not belong on the same level. [...]. And putting activities and participation on the same level once again is a barrier. Because it's the question [...]: What are we looking at, can do or do?”* [FG4_physician_31y experience]

#### Adaptability

The degree to which an innovation can be adapted, tailored, refined, or reinvented [38, 63]. The possibility and requirement to adapt the ICF for different settings within the profession and the multidisciplinary team, were mainly discussed in the two multidisciplinary groups. Practical examples of how the ICF has been adopted in interdisciplinary settings and that it would be necessary to integrate dietitians in this team and how to integrate them were stressed. (**Facilitator)**

*“I could imagine expanding our target catalogue to include such [dietetic] goals.”* [FG4_linguist_16y experience]

#### Trialability

The ability to test the innovation on a small scale in the organization, and to be able to reverse course if warranted [38, 63]. Discussants mentioned that it would be helpful to test the assignment to specific ICF categories on a low level prior to the implementation of the whole classification system. **(Facilitator)**

*“[...] and the colleagues now, for example, [...] once so that they can give it a try, [...]. Not with pressure, [...] without the complex ICF system behind it.”* [FG2_director_36y experience]

#### Complexity

Perceived difficulties of the innovation [38, 63]. Enough time to practice was seen as a promotive factor to integrate the new terminology in clinical dietetic practice. **(Facilitator)**

*“[...] if you practice that [...] then that's quite possible.”* [FG1_dietitian_11y experience]

Mentioned were the difficulty of a proper assignment of intervention goals to an appropriate ICF category, as well as the use of Environmental and Personal Factors. Furthermore, several concepts were not easy to understand and needed a revision. The great amount of ICF categories and especially of the added specific dietetics categories and the effort to implement a standardized process were considered in all four focus groups. **(Barrier)**

*“[...] because of the convolution, there is too much in there I find, so more for physical therapists or occupational therapists.”* [FG1_dietitian_2y experience]; *“[...] and that's actually the biggest barrier I see [...] expanding and ultimately increasing the amount* [in additional ICF-Dietetics categories] *and the complexity.”* [FG4_physician_31y experience]

#### Design Quality & Packaging

Perceived excellence in how the innovation is presented, and assembled [38, 63]. As main facilitator, the application concept and the web-based search function were emphasized. Additionally, the integration into electronic health records was discussed as prerequisite for a successful implementation. In this context, having a table of contents and a Core Set with the most typical ICF-Dietetics categories were considered. In the pretest focus group, participants reported that several ICF categories should be checked for their necessity. **(Facilitator)**

*“But as I said, if you had tools like Core Sets or something, it would definitely be helpful. Very helpful.”* [FG1_dietitian_2y experience]

The challenging endeavor to find a balance between completeness and clarity, was mentioned as an obstacle. **(Barrier).**

### CFIR domain outer setting

#### Needs & resources of those served by the organization

The extent to which the needs of those served by an organization (e.g., patients) are accurately known and prioritized by the organization [38, 63]. No patient participated in any of the focus group, however, advantages for patients were addressed in all groups, for example the increased involvement of patients in the therapeutic process, the possibility to receive a more individualized therapy and continuity of care, as well as a better understanding of the findings and therapeutic goals by patients. **(Facilitator)**

*“[...] also involving the person concerned. It's also a win for him, if he really has a clear dietetics diagnosis [...].”* [FG1_dietitian_11y experience]

Using the ICF explicit for a single professional group (e.g. for dietitians) was seen as problem for patients and should be carefully considered. **(Barrier)**

*“[...] if you want to use the ICF idea to break up [the deficit orientation] and focus on the patient rather than the profession, [...], I think it’s very problematic, to introduce profession-specific things. [...] I do not object using it, just be aware that you are not losing, in a way, the basic goals of the whole, namely a stronger focus on participation, and on the activities.”* [FG3_physician_30y experience]

#### Peer pressure

Mimetic or competitive pressure to implement an innovation [38, 63]. The fact that in the future it will be required to justify dietetic interventions against hospital association or social insurance institutions was an issue that was discussed as facilitator. **(Facilitator).**

As a great barrier in terms of difficulties in implementing the ICF the prevailing deficit-oriented health care was emphasized from one participant of a multidisciplinary group. **(Barrier)**

*“[...] so we are extremely deficit-oriented and have been drilled in our training always deficit-oriented. And I think that this deficit-orientation is probably also the biggest barrier to coming to a simple language* [like the ICF]*.”* [FG4_physician_31y experience]

#### External Policy & Incentives

This construct includes e.g. external strategies to spread innovations including policy and regulations, as well as public or benchmark reporting [38, 63]. Participants, especially in the program directors focus group, were convinced that it requires external policy and best practice examples to implement a standardized terminology. **(Facilitator)**

*“[...] that in some cases best practice examples would be needed.”* [FG1_dietitian_2y experience]*; “Maybe some way will then go through the Ministry, with the Quality Assurance Department [...].”* [FG2_director_30y experience]

### CFIR domain inner setting

#### Networks & Communications

The nature and quality of formal and informal communications within an organization [38, 63]. In both multidisciplinary focus groups the importance of communications between all health professionals was discussed and practical examples were given on how communication within the interdisciplinary team in the inner setting of the institution could work. **(Facilitator)**

*“So, we have an IT-technical network, where all professional groups see, if you* [to the speech therapist] *change something.”* [FG3_dietitian_18y experience]

One expert mentioned that using the ICF explicit for a single professional group is a great barrier not only for patients as described above but also for inter-professional communication. **(Barrier)**

*“[...] If that is not the standardized language of the house at the same time. If the chef does not know what that is. If the nurse does not know exactly what it is. [...] the problem of a profession-specific language is that it immediately starts to become problematic if it is not permeable to other professions. [...].”* [FG3_physician_30y experience]

#### **Implementation climate** (four sub-constructs were discussed)

The capacity for change, shared receptivity of involved individuals to an innovation, and the extent to which use of that innovation will be supported within the organization [38, 63]. It was emphasized that a successful implementation had to be supported from the management and leadership of the institutions. **(Facilitator)**

*“But I now believe that it is the pressure in the houses that is needed, [...] that is, the pressure from above.”* [FG2_director_26y experience]

ICF-Dietetics fits in the dietetics care process, which was seen as advantage. The dietetic diagnosis finding process and the possibility for using ICF-based assessment instruments were positively mentioned. **(Facilitator).**

Rewards were discussed to be a facilitator while lacking rewards were seen as barrier. **(Facilitator and Barrier).**

One expert mentioned, the turning away from current dietetic assessments toward an assessment according the ICF components under the terms of the ICF model as a main barrier for a successful implementation. **(Barrier)**

*“And if you say, okay, because of the classification you have to change the grammar* [current assessment practice]*, that's a huge barrier. Nobody will want to go there.”* [FG4_physician_31y experience]

#### **Readiness for implementation** (two sub-constructs were discussed)

Tangible and immediate indicators of organizational commitment to its decision to implement an innovation [38, 63]. Participants mentioned that managers and the leadership of an institution had to embrace the implementation of the ICF-Dietetics in order to achieve successful implementation. **(Facilitator)**

*“[...] that has to be supported in the house, also by their structures. [...] I believe that this must be anchored in the quality management of a house, so that it then also will be integrated in the documentation systems.”* [FG2_director_26y experience]

A lack of involvement and commitment by the leadership was seen as a barrier to successful implementation. In that context, the financing of the implementation, additional time and personal resource were addressed as risks for the implementation. **(Barrier)**

*“The institutions do not want that, because then their own staff needs even more documentation time.”* [FG3_physician_30y experience]

### CFIR domain characteristics of individuals

#### Knowledge & Beliefs about the innovation

Individuals’ attitudes toward and value placed on the innovation, as well as familiarity with facts, truths, and principles related to the innovation [38, 63]. The fact that was often mentioned was the importance of persuasion, especially, to convey the advantages of implementing the ICF-Dietetics within a multiprofessional approach, of the usability of the concept and that some criteria were already met by the current dietetic work. **(Facilitator)**

*“[...] Yet, I believe that one would have to do a lot of persuading and also the advantages for the individual dietitian also should be worked out, so that this will be widely accepted.”* [FG1_dietitian_2y experience]*; “That means, if you want to make it attractive for dietitians, then it is necessary that you really convey this concept and then convey the usability of the concept*.” [FG4_physician_31y experience]

#### Other personal attributes

Other personal traits such as tolerance of ambiguity and motivation [38, 63]. The risk of stakeholders refusing the implementation of the ICF-Dietetics, because it is something new, was seen as an obstacle. **(Barrier)**

*“But of course, it’s a change and that will certainly be provoking resistance at the beginning [...], as with all new stuff.”* [FG1_dietitian_11y experience]

### CFIR domain process of implementation

#### Planning

A construct about scheme or method for implementing an innovation [38, 63]. The majority of the participants considered a stepwise implementation process (bottom-up) to be preferable. **(Facilitator)**

*“Yes, so I join in too. I also think it's easier to take small steps.”* [FG3_nurse_4y experience]*; “Serve the concept in healthy, tasty titbits.”* [FG4_linguist_16y experience]

Although some advantages for a top-down implementation were pointed out, more disadvantages were seen. **(Barrier)**

*“A top-down process [...] certainly has its advantages, but the big disadvantage is that you have to realize it, as it were [...] it has to be succeeded.”* [FG3_physician_30y experience]

#### Engaging

Involving appropriate individuals in the implementation and use of the innovation through a combined strategy of social marketing, education, and similar activities [38, 63]. In this regard, the participants mentioned that it would be necessary to combine different strategies in the implementation, such as conducting trainings in the use of the ICF-Dietetics and the application concept for practicing dietitians and supervisors for interns on the one hand and for teachers of the universities on the other hand. The trainings have to be very practical and consistent among different providers. Furthermore, the concept has to be integrated in the curriculum of universities. (**Facilitator)**

*“I think it also needs very specific training to be able to really apply that. It should also be integrated in the curriculum, in the universities, that it needs a special focus to learn gradually.”* [FG2_director_40y experience]

#### Opinion leaders

Individuals in an organization that have formal or informal influence on the attitudes and beliefs of their colleagues with respect to implementing the innovation [38, 63].

In a multidisciplinary group the advantage of an opinion leader was addressed. **(Facilitator)**

*“And it really needs someone in a house who says: well, we'll do that now [...]. And then it takes a team that moves along, but it needs someone [...] as driving force.”* [FG4_linguist_16y experience]

### Linked interventions, theories and responsibilities

The theory-based approach for linking focus group data and interventions was found to be valid by the subsequent applied group process. Results in terms of linked interventions, the underlying theories and the responsibilities in terms of responsible stakeholders according to intervention objectives and its context requirements is shown in Table 4. The interventions were allocated to five main responsible stakeholders (beside the engagement of institution leadership), namely the Association of Dietitians, the developer of the ICF-Dietetics application concept, potential implementation leaders, the program directors of universities of applied sciences and clinical dietitians as key stakeholders.

**Table 4:** Results in terms of linked interventions, underlying theories and responsibilities based on implementation criteria according to CFIR domains/constructs

CFIR Domain	CFIR Construct	Implementation strategy criteria (points to consider based on focus groups)	Intervention (based on focus groups and literature [39, 40, 43, 44, 60–62])	Underlying theory [27, 39, 40]	Responsibility
Intervention characteristics	Evidence Strength & Quality	Starting implementation with pilot institutions. Adducing ICF-Dietetics field studies and other examples (e.g. nursing language).	Conduct a Pilot study		Research Institution
	Relative Advantage	Conveying the benefits of ICF-Dietetics.	Provide information: on the ICF-Dietetics its advantages and disadvantages adapted to different learning styles	Cognitive theory on learning	Association of Dietitians
	(disadvantage)	Considering the drawbacks of using the ICF in form of a professional-specific terminology. Avoiding over-categorizing.	Provide information for other professional groups Refine application concept to improve attractiveness	Theory on organizational learning, Theory on learning	Implementation leader^1^ Developer of the concept^2^
	(advantage and disadvantage)	To consider if the ICF model should be introduced in education and practice.	Refine application concept to improve attractiveness	Theory on learning	Developer of the concept
	Adaptability	The application concept and the ICF-Dietetics (granularity) have to be adaptable to different settings and workflows in professional practice. In principle, the ICF / ICF-Dietetics offers this possibility.	Provide information: on the possibility for adaptability to different settings adapted to different learning styles Enable self-regulation to adapt application to individual needs	Cognitive theory on learning Behavior, observational learning	Association of Dietitians Developer of the concept
	Trialability	Providing examples for practice purposes before implementation.	Start with practical-related interactive workshops before implementation	Social cognitive theory	Association of Dietitians
	Complexity	Being aware of barriers of complexity. It takes extensive experience regarding the assignment to appropriate ICF-Dietetics categories, the use of qualifiers, as well as the assignment to environmental factors and personal factors.	Start with practical-related interactive workshops before implementation Provide training to change group processes	Social cognitive theory Theory on team effectiveness, group decisions	Association of Dietitians (ICF trainers) Implementation leader
			Provide skills training and feedback on performance	Cognitive theory on learning	Implementation leader
			Enable self-regulation to adapt application to individual needs	Behavior, observational learning	Developer of the concept
		Putting codes in the background.	Refine application concept to improve attractiveness	Theory on learning	Developer of the concept
		Recognizing the large number of ICF-Dietetics categories as a major barrier. Being aware of the need to develop a nutrition and dietetics-related Core Set.	Refine application concept to improve attractiveness: Develop a nutrition and dietetics-related Core Set	Theory on learning	Developer of the concept or Researcher
		Being aware of the barrier of initial effort.	Define individual goals for change	Motivational theories	Implementation leader
		Taking into account and communicate the need of additional time especially, at the beginning.	Provide training to change group processes	Theory on team effectiveness, group decisions	Implementation leader
		Perceived incompleteness of the ICF-Dietetics categories may come through lack of practice and experience in the use of the new language.	Involve opinion leaders or professional peers (educational outreach)	Theories of planned behavior and social comparison	Implementation leader
	Design Quality & Packaging	There is a need for an intelligent search function, and the integration of the ICF-Dietetics in electronic health record systems.	Incorporate the ICF-Dietetics into existing information systems for coding purposes	Theory on organizational learning	Implementation leader or institution leadership
		The application concept has to be well designed and clear. Clarifying questions, such as; what should be documented? What should be done with the documentation?	Refine application concept to improve attractiveness	Theory on learning	Developer of the concept
		Beginning with a small Core Set, that should be extensible.	Refine application concept to improve attractiveness: develop a nutrition and dietetics-related Core Set	Theory on learning	Developer of the concept
		Beginning with a simplified application.	Refine application concept to improve attractiveness	Theory on learning	Developer of the concept
		There is a need for a table of contents.	Refine application concept to improve attractiveness	Theory on learning	Developer of the concept
		There is a need for a revision of the ICF-Dietetics (in cooperation with the proprietors of the original ICF-Dietetics).	Refine application concept to improve attractiveness: Revise the ICF-Dietetics	Theory on learning	Developer of the concept
		There is a need for a balance between completeness and not confusing.	Refine application concept to improve attractiveness: Revise the ICF-Dietetics	Theory on learning	Developer of the concept
Outer setting	Needs & Resources of Those Served by the Organization	Focusing on patient orientation and patient goals and continuing of care. Recognizing that the focus only on interventions goals, that has set by health professionals could be a great barrier in terms of patient-centered care.	Take care of patient focused goals and satisfaction	Theory on quality management	Dietitians
	Peer Pressure	Conveying the awareness of the necessity to ensure evidence in the future.	Provide general information: to ensure evidence	Cognitive theory on learning	Association of Dietitians
	External Policy & Incentives	The implementation of the ICF-Dietetics nation-wide should be supported by politics and legal regulation.	Influence decision makers, build political support	Theory on agenda building	Association of Dietitians
		Presentation of the concept at congresses and other health care events.	Influence decision makers, build stakeholder support	Theory on agenda building	Association of Dietitians
		The recently started realization of Primary Health Care Centers could be facilitate the implementation of a multidisciplinary applicable terminology.	Influence decision makers, build public support	Theory on agenda building	Association of Dietitians
		Publishing best practice examples.	Provide information of best practice examples	Cognitive theory on learning	Association of Dietitians
Inner setting	Networks & Communications	Integrating and inform other health care professional and aiming a common solution.	Make better use of information technology Provide information for other professional groups	Theory on organizational learning Theory on integrated care	Implementation leader or institution leadership
	Tension for Change	Necessity for implementation have to come from leadership of institutions.	Provide specific information on the advantages of the ICF-Dietetics for managers and the leadership of the institution	Theories on persuasion and leadership	Implementation leader
		Tension for change has to be seen and build up within the professional group.	Implement continuous improvement activities	Theories on quality management	Implementation leader or institution leadership
	Compatibility	ICF-Dietetics needs to be adapted to the dietetic care process, not the other way around. The ICF is not an assessment, but for developing assessments for functioning.	Implement continuous improvement activities	Theories on quality management	Implementation leader or institution leadership
			Create teams/collaborative for improvement	Theories on quality management	Implementation leader or institution leadership
			Recruit and train leaders to integrate or establish a continuous improvement program in dietetics care	Theories on quality management	Implementation leader or institution leadership
			Enable self-regulation to adapt application to individual needs	Behavior, observational learning	Developer of the concept
	Relative Priority	Conducting needs assessment before implementation, e.g., about the perceived importance of implementing a standardized terminology in dietetics.	Not necessary: evaluations have already been conducted		
	Organizational Incentives & Rewards	There should be a defined compensation of the additional required time and the recognition from the leadership of the institutions.	Define compensation of the additional required time and the recognition from the leadership of the institutions	Reimbursement theories	Implementation leader or institution leadership
	Leadership Engagement	Management and leadership of institutions (e.g. the quality assurance departments) have to take responsibility for the implementation.	Provide specific information on the advantages of the ICF-Dietetics for managers and the leadership of the institution	Theories on persuasion and leadership	Implementation leader
	Available Resources	Resources, especially time and/or additional human resources, have to be clarified in advance.	Provide information about additional resources and clarify them in advance	Theories of Leadership	Implementation leader
Characteristics of individual	Knowledge & Beliefs about the Innovation	Conveying clear usability of the application concept and the ICF-Dietetics, e.g. how it works and which steps and ICF-Dietetics categories should be documented.	Provide general information Involve opinion leaders or professional peers (educational outreach)	Cognitive theory on learning Motivational theories	Association of Dietitians Implementation leader
		Conveying the usability of the ICF-Dietetics within a multiprofessional approach, and conveying that not everything is new, but has already been applied in dietetic practice.	Provide general information Involve opinion leaders or professional peers (educational outreach)	Cognitive theory on learning Motivational theories	Association of Dietitians Implementation leader
	Other Personal Attributes	Motivating dietitians in order to prevent resistance, e.g. motivate them to overcome the first needed effort for a higher aim.	Define individual goals for change	Motivational theories	Implementation leader
Process of implementation	Planning	Evaluate what is taught at universities regarding standardized terminologies in general and about the ICF in particular.	Not necessary: all program directors of universities participated in the focus groups		
		Planning the implementation stepwise (e.g., firstly, standardizing the assessments and the dietetics diagnosis, then adopting intervention goals with pre-defined goal lists in terms of ICF-Dietetics categories).	Apply intervention stepwise according to the “stage” of change	Stages-of-Change Theories	Dietitians
		Standardizing the dietetic care process that is taught in universities.	Standardize teaching plans of dietetics universities	Theory on learning	Directors of universities
		Further validation of the ICF-Dietetics should be done in the ongoing process.	Refine application concept to improve attractiveness: Revise the ICF-Dietetics	Theory on learning	Dietitians and developer of the concept
	Engaging	Offering of trainings and ICF workshops for practicing dietitians, supervisors for interns and teachers.	Start with practical-related interactive workshops before implementation Provide continuous trainings	Social cognitive theory Theory of Total Quality Management	Association of Dietitians Implementation leader
		Developing practice-oriented standardized training material.	Provide printed educational material, e.g. a manual of the dietetics care process and the use of the ICF-Dietetics	Theory on learning	Association of Dietitians
	Opinion Leaders	Institutions need a person as an opinion leader.	Involve opinion leaders or professional peers (educational outreach)	Theories of planned behavior and social comparison	Implementation leader
	(Key Stakeholders) ^3^	Addressing different settings and work experience of dietitians, such as, students, freelancers and employees, those they just finished their education and those who have been in practice for many years.	Provide information on the ICF-Dietetics for adaptability to different settings adapt to different learning styles	Cognitive theory on learning	Association of Dietitians

^1^The implementation leader is someone who is the champion on each facility usually the leading dietitian

^2^The developer of the concept is the researcher who has develop the application concept to integrate the ICF-dietetics in the Austrian dietetic care process

^3^The construct key stakeholder is described in the CFIR codebook however not mentioned as separate CFIR construct by Damschroder et al. [38]

### The logic model for the ICF-dietetics implementation

**Fig. 3:**
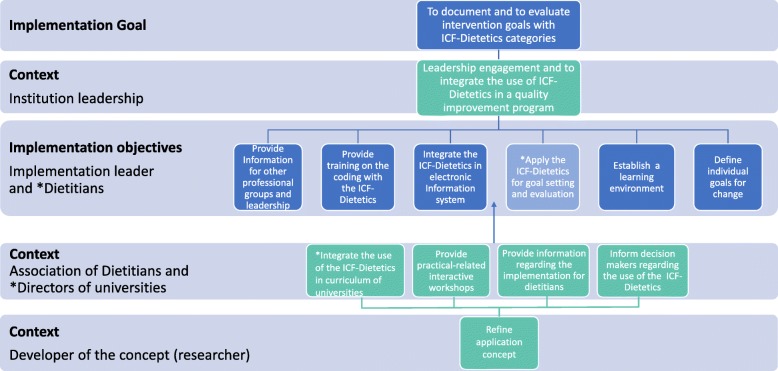
Logic model for the ICF-Dietetics implementation. This logic model was developed by merging results of linked interventions according to its responsibilities which are shown in Table 4

The logic model, shown in Fig. 3, summarizes the interventions with respect to its responsibilities. The implementation criteria were assigned to six implementation objectives with the responsibility of intervention leaders and dietitians, respectively: (1) provide information for other professional groups, (2) provide training on the coding with the ICF-Dietetics to change group processes, (3) integrate the ICF-Dietetics in electronic health information system and into all forms of documentation, (4) apply the ICF-Dietetics for goal setting and evaluation stepwise to all consultations, (5) establish a learning environment in terms of skills training and feedback on performance by an opinion leader or professional peers, as well as (6) defining individual goal for change. In addition, the implementation criteria were mapped to six context requirements at the following three levels of responsibilities: (1) developer of the ICF-Dietetics application concept (researcher and first author of this study), (2) the Association of Dietitians and directors of universities and (3) institution leadership.

## Discussion

This study explored the clinical practicability and acceptability of the ICF-Dietetics along with barriers and facilitators to its nationwide implementation. The study was conducted prior to the implementation focusing on facilitators and barriers of a future implementation, since literature has shown that tailored implementation strategies increases the likelihood of successful dissemination and implementation [42, 47, 51, 72]. Merging our results, we established a logic model for the nationwide implementation of the ICF-Dietetics.

In general, the participants of the pretest agreed that the ICF-Dietetics would be useful and acceptable for clinical practice. Compared to the previous study of mapping clinical care report concepts to the ICF-Dietetics [10], the pretest showed that numerically more ICF-Dietetics categories (especially in the ICF component activities and participation as well as environmental factors) were applied (248 compared to 153, respectively) in fewer documentations (55 compared to 100, respectively). This could be related to the fact that the dietitians were trained in the use of the ICF-Dietetics in the pretest study. As it was emphasized by participants of the pretest, the use of the ICF-Dietetics with its underlying biopsychosocial model helps to gain additional information that otherwise has been missed. We concluded that the ICF-Dietetics widens the perspective of dietitians. Furthermore, the pretest confirms all but one of the most frequently assigned ICF-Dietetics categories as described in the previous study [10] and added 22 more second level ICF-Dietetics categories to the list of ICF-Dietetics categories important for nutrition and dietetics that has been revealed by previous studies [10, 11]. Thus, the final list consists of 160 second level ICF-Dietetics categories out of exactly 2402 ICF-Dietetics categories and 422 second level categories, respectively. This list of ICF-Dietetics categories could be used as basis for developing so called ICF-Dietetics Core Sets, which are purpose tailored short lists of ICF categories relevant for patients with a certain health condition [10].

Furthermore, participants of the pretest assumed the educational program to be sufficient enough for this pretest aiming at documenting the most important ICF-Dietetics categories for setting intervention goals based on the assessment and the dietetics diagnosis. However, considering 6 h of training might not be enough for an accurate and comprehensive use of a new standardized terminology for clinical dietetic care. They need more concrete examples to practice the application with feedback from others. Therefore, for a nationwide implementation of the ICF-Dietetics, more practice-related workshops have to be provided preferably before the implementation is rolled out. Moreover, the application was perceived in several parts as difficult. On the one hand, this was due to the fact that a new concept had to be applied, on the other hand, that the application concept developed for this project still needed to be improved. This is in line with literature regarding effective implementation of change in patients’ care, e.g. change in clinical care practice is optimally achieved using well-designed intervention [47] and presentation that is perceived as easily understandable by the professionals who would be affected by the planned change [38].

As main barrier, the complexity of the ICF-Dietetics was discussed in the focus groups. In this regard, the high number of about 2400 categories and the consequences of this, namely difficulties in assigning the appropriate one, has to be mentioned. This finding was confirmed by an earlier ICF implementation study in Germany [73]. However, the issue of complexity was also reported in implementing the NCP/NCPT [20]. Nevertheless, this barrier needs to be carefully targeted during the implementation. Otherwise, comparability would be substantially affected hence some categories were maybe more easily identified than others and the assignment could depend on users. Advantages of a more stringent and logical re-definition of the ICF categories in general have also been reported by Heerkens et al. [74], such as reduction of ambiguity of concepts and improvement of ICF use efficacy that would lead to better semantic interoperability. Moreover, information, training and education of the professionals who are going to use the ICF-Dietetics, might be a possible strategy to overcome the complexity of difficulties regarding the number of categories and their assignment. Training was not only an important topic in our study, but also found to be a critical success factor in other studies [18, 20, 73]. It has to be mentioned, not every education is effective in changing practice [47, 71]. For successful implementation strategy, interactive and continuous education, including discussion of evidence and feedback on performance [19, 47, 53] or educational outreach visits [52] are needed.

The web-based search function that had been provided for the pretest was seen as great advantage in usability. Additionally, a highly-recommended improvement before implementation in a larger scale, the integration of the ICF-Dietetics in electronic health record system was discussed as imperative, and confirmed by other studies [13, 75]. Rossi et al. [13], for instance, have shown in terms of implementation of the NCP and NCPT in a Single-Center Hemodialysis Unit, significant improvements in the efficiency of nutrition care and effectiveness related to patient outcomes of an electronic versus a manual paper-based nutrition care documentation system. Vreeman and Richoz [75] have pointed out that incorporating ICF and other internationally accepted standardized terminologies and aid tools into clinical information systems, assist the clinical decision-making thus advance clinical practice and research by enabling data sharing.

Regarding the implementation process the majority of focus group participants in our study emphasized that a stepwise (bottom-up) approach would be preferable compared to a top-down approach. For example, focus group participants mentioned, firstly, standardizing the assessment and the dietetics diagnosis step, secondly, adopting intervention goals with pre-defined goal lists in terms of ICF-Dietetics categories that can be evaluated. The major concern regarding the top-down approach discussed by one participant was the implementability nationwide. Taking all focus group discussions into consideration, a combined strategy would be necessary, namely, encouraging and empowering people to achieve change in their range of influence on the one side, providing central coordination of efforts from leadership of institution, politics and the association of dietitians on the other side. This approach is in line with Ham et al. [76], who have indicated, that the role of organizations at different levels needs to be addressed as part of an integrated and coherent quality improvement strategy. The important roles and interrelationships of leadership engagement and available resources have been discussed in the focus groups and shown in literature [45, 77], for instance, the leadership engagement can lead to provision of sufficient available resources in terms of dedicated time. Consequently, the leadership engagement and integration of the ICF-Dietetics in a quality improvement program will be crucial and a prerequisite for a successful nationwide implementation.

Moreover, results of the focus groups showed the importance of facilitating policy and external incentives as potential context factors for the implementation of a standardized terminology which is also confirmed in literature [38, 57, 78]. Therefore, lobbying with decision makers regarding the use of the ICF-Dietetics by the Association of Dietitians might be essential. While training or educational strategies typically target knowledge and skills, financial and policy strategies enhance fidelity and acceptance [57] and both play an important role when implementing a standardized terminology.

### Strengths and limitations

Identified criteria (points to consider) in terms of barriers and facilitators could be linked to interventions and responsibilities applying a structured framework to inform a targeted nationwide implementation strategy. Using the codebook of the CFIR provided us with constructs and definitions for organizing qualitative data. The main benefits of this approach, such as to have an a priori templates, is that it accelerates the coding process and generates comparable results. The first coding and developing intervention-specific themes helped us to overcome the disadvantages of attending predefined constructs, namely, of missing important aspects. From our findings in terms of in-depth interviews, we conclude that focus groups were a suitable method to identify barriers and facilitators regarding the implementation of a standardized terminology. We found, that focus groups allowed the participants to discuss and question perspectives of colleagues, that, in turn raised some more important perspectives that could be investigated in addition, for example in terms of Core Set development and assessment instruments.

The pretest was necessary to evaluate the practicability of the application concept for integrating the ICF-Dietetics in clinical practice. The aims were foremost to validate the developed application concept and to inform the subsequent focus groups. Due to the wide range of application possibilities of the ICF-Dietetics and the about 2400 categories a standardized application concept is a requirement for standardized use of the ICF-Dietetics. Otherwise, as discussed above, comparability would be substantially affected.

A limitation of the pretest was that the results are restricted to one institution and to only four of 22 dietitians who applied the ICF-Dietetics in their clinical practice. Therefore, the results of the pretest are not generalizable for other settings. Moreover, these four dietitians who volunteered for participating in our study could have had in principle a more positive attitude towards implementing the ICF-Dietetics when compared to their colleagues. A generally positive attitude may be a facilitator in implementing the ICF-Dietetics in clinical practice in itself. No reason of not participating was asked, we assume as possible reasons time constraints and lack of interest. This could reflect the general interest and attitude of implementing a standardized terminology in clinical practice. To generate interest in a common new terminology, information and activities that lead towards a positive attitude would be essential in a successful implementation strategy. Individual goals for change as well as incentives may further help to motivate dietitians [36].

A further limitation is our sampling approach of selecting extreme cases which affects generalizability of focus group results. Choosing the four dietitians who took part in the pretest and multidisciplinary groups that are already applying the ICF might have caused a selection bias due to missing opinions of ICF-Dietetics opponents. However, we applied this method to get in-depth insight of a specific group of experts that were acquainted with advantages and disadvantages of the ICF usage in clinical practice for informing an implementation strategy.

## Conclusions

The results of this study demonstrated that for a successful nationwide implementation of the ICF-Dietetics, several specific criteria concerning the ICF-Dietetics and the application concept should be addressed and specific interventions need to be applied by different stakeholders. These results set the foundation for developing a targeted implementation strategy to increase the success, reproducibility and comparability.

## Supplementary information

**Additional file 1.** Documentation tools.

**Additional file 2.** Interview guide for focus groups.

**Additional file 3.** List of applied second-level ICF-Dietetics categories in respect of medical areas.

**Additional file 4.** Implementation criteria according to CFIR domains/constructs and identified higher level-themes.

## Data Availability

Most of the data generated or analyzed during this study are included in this published article [and its supplementary information files]. The original transcripts and entire original framework analysis matrix (both in German language) are available from the corresponding author on reasonable request.

## Abbreviations

CFIR: Consolidated Framework of Implementation Research
DCP: Dietetic care process
ICF-Dietetics: International Classification of Functioning, Disability and Health for Dietetics
NCP: Nutrition care process
NCPT: Nutrition care process terminology
