# Supplementary material for: Towards a nationwide implementation of a standardized nutrition and dietetics terminology in clinical practice: a pre-implementation focus group study including a pretest and using the consolidated framework for implementation research
Source: BMC Health Serv Res. 2019 Nov 29;19:920. doi: 10.1186/s12913-019-4600-5 (PMC6884883; doi:10.1186/s12913-019-4600-5)
Supplement: Supplementary file 1 — Additional file 1. Documentation tools. [file 12913_2019_4600_MOESM1_ESM.docx]

**Additional file 1.** Documentation tools

These tools (templets for the documentation of the nutrition and dietetic assessment, diagnosis and goal setting) based on the ICF Research Branch (25), Rauch et al. (24) and Gäbler et al. (10). First part depicts the ICF-Dietetics Assessment Sheet (reflecting the patient and health professional’s perspective), the second part the ICF-Dietetics Categorical and Goal Profile (as a visual depiction of a patient's functioning status in terms of ICF-Dietetics categories).
