# Supplementary material for: Towards a nationwide implementation of a standardized nutrition and dietetics terminology in clinical practice: a pre-implementation focus group study including a pretest and using the consolidated framework for implementation research
Source: BMC Health Serv Res. 2019 Nov 29;19:920. doi: 10.1186/s12913-019-4600-5 (PMC6884883; doi:10.1186/s12913-019-4600-5)
Supplement: Supplementary file 2 — Additional file 2. Interview guide for focus groups. [file 12913_2019_4600_MOESM2_ESM.docx]

**Additional file 2.** Interview guide for focus groups

Opening: We start the discussion by introducing yourselves; what is your health profession, how long do you work in your profession, what are your areas of expertise and what are your experiences regarding the ICF?

Presentation: ICF/ICF-Dietetics and application concept

Interview with semi-structured questions:

- Please introduce and discuss your perspectives on ICF-Dietetics as a derived version of the ICF after you have listened to this presentation.
- What advantages do you see in the application of the ICF/ICF-Dietetics?
- What disadvantages do you see?
- What, in your opinion, are the difficulties, and risks that can occur when the ICF-Dietetics will be introduced in clinical dietetic practice? Within the institutions and outside.
- What opportunities and strengths do you see? Within the institutions and outside.
- What are the prerequisites (what must exist) for the implementation of ICF-Dietetics in clinical practice?
- (optional) What are prerequisites (what is important) for the use of ICF-Dietetics in multidisciplinary collaboration?
- What was important for you in our discussion? Please summarize again briefly the most important aspects?
