# Supplementary material for: Towards a nationwide implementation of a standardized nutrition and dietetics terminology in clinical practice: a pre-implementation focus group study including a pretest and using the consolidated framework for implementation research
Source: BMC Health Serv Res. 2019 Nov 29;19:920. doi: 10.1186/s12913-019-4600-5 (PMC6884883; doi:10.1186/s12913-019-4600-5)
Supplement: Supplementary file 3 — Additional file 3. List of applied second-level ICF-Dietetics categories in respect of medical areas. [file 12913_2019_4600_MOESM3_ESM.docx]

**Additional file 3**. List of applied second-level ICF-Dietetics categories in respect of medical areas

| ICF-Code | ICF-Dietetics categories | Diabetes  Metabolic diseases | Gastro-enterology | Surgery | Onco-logy | Others ^a^ |
| --- | --- | --- | --- | --- | --- | --- |
|  | **BODY FUNCTIONS** |  |  |  |  |  |
| b117 | Intellectual functions |  |  |  |  | X |
| b126 | Temperament and personality functions |  |  | X | X |  |
| b130 | Energy and drive functions | X | X | X | X | X |
| b140 | Attention functions | X | X | X | X |  |
| b144 | Memory functions |  |  |  |  | X |
| b152 | Emotional functions |  | X | X | X |  |
| b160 | Thought functions |  | X | X |  |  |
| b164 | Higher-level cognitive functions | X | X | X | X | X |
| b167 | Mental functions of language | X |  | X | X |  |
| b210 | Seeing functions |  |  | X |  |  |
| b230 | Hearing functions |  |  |  |  | X |
| b250 | Taste function |  | X |  | X |  |
| b280 | Sensation of pain | X | X | X | X |  |
| b420 | Blood pressure functions | X |  | X |  |  |
| b431 | Clinical chemical blood composition | X | X | X | X | X |
| b433 | Hematological blood characteristics | X |  |  | X |  |
| b435 | Immunological system functions |  |  | X |  |  |
| b440 | Respiration functions |  |  |  | X |  |
| b450 | Additional functions of the respiratory system |  |  |  |  | X |
| b510 | Ingestion functions | X | X |  | X |  |
| b515 | Digestive functions |  | X | X | X | X |
| b525 | Defecation functions |  | X | X | X |  |
| b527 | Other functions related to defecation |  |  | X |  |  |
| b531 | Weight change | X | X | X | X |  |
| b532 | Nutritional status |  |  |  | X |  |
| b533 | Functions of structures related to digestion | X | X | X | X | X |
| b534 | Body composition |  | X |  | X | X |
| b535 | Sensations associated with the digestive system |  | X | X | X |  |
| b540 | General metabolic functions |  |  | X | X | X |
| b545 | Water, mineral and electrolyte balance functions | X |  |  |  |  |
| b555 | Endocrine gland functions | X |  |  |  |  |
| b610 | Urinary excretory functions | X |  | X |  | X |
|  | **BODY STRUCTURE** |  |  |  |  |  |
| s320 | Structure of mouth | X |  |  | X |  |
| s530 | Structure of stomach |  |  | X |  |  |
| s540 | Structure of intestine |  |  | X | X |  |
| s560 | Structure of liver |  | X |  |  |  |
| s705 | Anthropometrics | X | X | X | X | X |
|  | **ACTIVITIES AND PARTICIPATION** ^b^ | | | | | |
| a133 | Acquiring additional language | X |  |  |  |  |
| a240 | Handling stress and other psychological demands | X |  | X |  |  |
| a398 | Communication, other specified |  |  |  |  | X |
| a4 | Mobility |  |  | X | X |  |
| a429 | Changing and maintaining body position,  other specified and unspecified | |  |  | X |  |
| a440 | Fine hand use | X |  |  |  |  |
| a460 | Moving around between different locations | X |  |  |  |  |
| a465 | Moving around using equipment | X |  |  |  |  |
| a520 | Caring for body parts | X |  |  |  |  |
| a530 | Toileting |  |  | X |  |  |
| a550 | Eating |  |  |  |  | X |
| a570 | Looking after one's health | X | X | X | X | X |
| a620 | Acquisition of goods and services | X |  |  |  |  |
| a630 | Preparing meals | X |  | X |  |  |
| a640 | Doing housework | X |  |  |  |  |
| p2 | General tasks and demands |  |  | X |  |  |
| p3 | Communication |  |  |  |  |  |
| p750 | Informal social relationships |  |  | X | X |  |
| p760 | Family relationships |  | X | X | X |  |
| p770 | Intimate relationships |  |  |  | X |  |
| p920 | Recreation and leisure | X | X | X |  |  |
|  | **ENVIRONMENTAL FACTORS** | | | | | |
| e110 | Products or substances for personal consumption | X |  | X |  | X |
| e165 | Assets | X | X |  |  |  |
| e310 | Immediate family | X | X | X | X | X |
| e320 | Friends | X |  |  |  |  |
| e340 | Personal care providers and personal assistants |  |  | X |  |  |
| e360 | Other professionals | X |  |  |  | |
| e355 | Health professionals | X |  | X |  |  |
| e410 | Individual attitudes of immediate family members |  | X |  |  |  |
| e620 | Work situation | X |  |  |  | X |
| e575 | General social support services, systems and policies | X | X |  |  |  |
|  | **PERSONAL FACTORS** ^c^ |  |  |  |  |  |
| pf410 | Personal effectiveness |  |  | X |  |  |
| pf450 | Compliance to therapy |  |  | X |  | X |
| pf465 | Knowledge of disease and functioning | X |  | X |  | X |
| pf610 | Smoking | X |  |  |  | X |
| pf625 | Use of medication |  |  |  |  | X |
| pf630 | Nutritional habits | X |  | X |  | X |
| pf645 | Hobbies |  |  | X |  |  |

^a^ Other medical areas included nephrology, pediatrics, neurology; ^b^ In contrast to the original ICF where “Activities and Participation” begins with (d), the ICF-Dietetics differentiates between “Activities (a)” and “Participation (p)” as it is also given as an alternative option by World Health Organization (58); ^c^ ICF-Dietetics provides a first draft of codes covering “Personal Factors (pf)”.
