# Supplementary material for: Towards a nationwide implementation of a standardized nutrition and dietetics terminology in clinical practice: a pre-implementation focus group study including a pretest and using the consolidated framework for implementation research
Source: BMC Health Serv Res. 2019 Nov 29;19:920. doi: 10.1186/s12913-019-4600-5 (PMC6884883; doi:10.1186/s12913-019-4600-5)
Supplement: Supplementary file 4 — Additional file 4. Implementation criteria according to CFIR domains/constructs and identified higher level-themes. [file 12913_2019_4600_MOESM4_ESM.docx]

**Additional file 4.** Results in terms of implementation criteria according to CFIR domains/constructs and identified higher level-themes

| **CFIR Domain** | **CFIR Construct** | **Higher-Level-Themes** | **Frequency^a^** | **Implementation strategy criteria (points to consider)** |
| --- | --- | --- | --- | --- |
| **Intervention characteristics** | **Evidence Strength & Quality** | Pilot Institution  Field studies  Comparisons | 2  1  8 | Starting implementation with pilot institutions. Adducing ICF-Dietetics field studies and other examples  (e.g. nursing language). |
|  | **Relative Advantage** | Alternatives  Multidisciplinary/ Interdisciplinarity  Comprehensibility  Comparability  Standardization  Information retrieval  Professionalization  Professional approach  Continuity of care  WHO terminology  Reimbursement  Holism | 3  13  1  4  5  7  10  14  3  3  2  6 | Conveying the benefits of ICF dietetics.  ICF-Dietetics has the great advantage of being a multidisciplinary, internationally known WHO terminology compared with alternatives such as NCPT or with developing a new dietetics language.  There are many other general advantages of ICF-Dietetics (e.g. comprehensibility, comparability, standardization, professionalization, etc.), that could facilitate implementation. |
|  | **(disadvantage)** | Interdisciplinarity/ Multidisciplinarity  Categorizing | 2  1 | Considering the drawbacks of using the ICF in form of a professional-specific terminology.   Avoiding over-categorizing. |
|  | **(advantage and disadvantage)** | Scheme of ICF model | 4 2 | To consider if the ICF model should be introduced in education and practice. |
|  | **Adaptability** | Dietetics  Nutrition support team  Research  Professional approach  Adaptability Granularity | 10  4  2  40  6  2 | The application concept and the ICF-Dietetics (granularity) have to be adaptable to different settings and workflows in professional practice.  In principle, the ICF / ICF-Dietetics offers this possibility. |
|  | **Trialability** | Trialability | 1 | Providing examples for practice purposes before implementation. |
|  | **Complexity** | Practice  Assignment  Qualifiers  Environmental  Personal Factors | 2  17  15  1  3 | Being aware of barriers of complexity.  It takes extensive experience regarding the assignment to appropriate ICF-Dietetics categories, the use of qualifiers, as well as the assignment to environmental factors and personal factors. |
|  |  | Code | 3 | Putting codes in the background. |
|  |  | Amount (in terms of complexity) | 12 | Recognizing the large number of ICF-Dietetics categories as a major barrier. Being aware of the need to develop a nutrition and dietetics-related core set. |
|  |  | Effort | 13 | Being aware of the barrier of initial effort. |
|  |  | Time (in terms of complexity) | 2 | Taking into account and communicate the need of additional time especially, at the beginning. |
|  |  | Incompleteness | 9 | Perceived incompleteness of the ICF-Dietetics categories may come through lack of practice and experience in the use of the new language. |
|  | **Design Quality & Packaging** | IT-support | 7 | There is a need for an intelligent search function, and the integration of the ICF-Dietetics in electronic health record systems. |
|  |  | Application concept | 7 | The application concept has to be well designed and clear. Clarifying questions, such as; what should be documented? What should be done with the documentation? |
|  |  | Core Sets | 7 | Beginning with a small Core Set, that should be extensible. |
|  |  | Simplification | 1 | Beginning with a simplified application. |
|  |  | Table of contents | 3 | There is a need for a table of contents. |
|  |  | Revision | 7 | There is a need for a revision of the ICF-Dietetics. |
|  |  | Amount (in terms of design) | 4 | There is a need for a balance between completeness and not confusing. |
| **Outer setting** | **Needs & Resources of Those Served by the Organization** | Patients  Professional approach (risk) | 2  1 | Focusing on patient orientation and patient goals and continuing of care. Recognizing that the focus only on interventions goals, that has set by health professionals could be a great barrier in terms of patient-centered care. |
|  | **Peer Pressure** | Evidence | 2 | Conveying the awareness of the necessity to ensure evidence in the future. |
|  | **External Policy & Incentives** | Politics | 9 | The implementation of the ICF-Dietetics nation-wide should be supported by politics and legal regulation.  Presentation of the concept at congresses and other health care events. |
|  |  | Primary Health Care | 3 | The recently started realization of Primary Health Care Centers could be facilitate the implementation of a multidisciplinary applicable terminology. |
|  |  | Best Practice Examples | 5 | Publishing best practice examples by the Association of Dietitians. |
| **Inner setting** | **Networks & Communications** | Interdisciplinarity/  Multidisciplinarity | 21 | Integrating and inform other health care professional and aiming a common solution. |
|  | **Tension for Change** | Institution (in terms of tension for change) | 1 | Necessity for implementation have to come from leadership of institutions. |
|  |  | Implementation (in terms of tension for change) | 1 | Tension for change has to be seen and build up within the professional group. |
|  | **Compatibility** | Application concept | 1 | ICF-Dietetics needs to be adapted to the dietetic care process, not the other way around. |
|  |  | Assessment instruments | 4 | The ICF is not an assessment, but for developing assessments for functioning. |
|  | **Relative Priority** | Priority | 1 | Conducting needs assessment before implementation, e.g., about the perceived importance of implementing a standardized terminology in dietetics. |
|  | **Organizational Incentives & Rewards** | Financing | 3 | There should be a defined compensation of the additional required time and the recognition from the leadership of the institutions. |
|  | **Leadership Engagement** | Institution | 4 | Management and leadership of institutions (e.g. the quality assurance departments) have to take responsibility for the implementation. |
|  | **Available Resources** | Resources | 4 | Resources, especially time and/or additional human resources, have to be clarified in advance. |

| **Characteristics of individual** | **Knowledge & Beliefs about the Innovation** | Persuasion | 5 | Conveying clear usability of the application concept and the ICF-Dietetics, e.g. how it works and which constructs should be documented.  Conveying the usability of the ICF-Dietetics within a multiprofessional approach, and conveying that not everything is new, but has already been applied in dietetic practice. |
| --- | --- | --- | --- | --- |
|  | **Other Personal Attributes** | Persuasion/Motivation | 4 | Motivating dietitians in order to prevent resistance, e.g. motivate them to overcome the first needed effort for a higher aim. |
| **Process of implementation** | **Planning** | Evaluation | 4 | Evaluate what is taught at universities regarding standardized terminologies in general and about the ICF in particular. |
|  |  | Implementation (in terms of planning the process) | 13 | Planning the implementation stepwise (e.g., firstly, standardizing the assessments and the dietetics diagnosis, then adopting intervention goals with pre-defined goal lists in terms of ICF-Dietetics categories).  Standardizing the dietetic care process that is taught in universities.  Further validation of the ICF-Dietetics should be done in the ongoing process. |
|  | **Engaging** | Training  ICF workshop  Education | 10  1  8 | Offering of trainings and ICF workshops for practicing dietitians, supervisors for interns and teachers.  Integrate ICF-Dietetics and application concept into education and adopting of the curriculum in regard to that.  Developing practice-oriented standardized training material. |
|  | **Opinion Leaders** | Driving force | 1 | Institutions need a person as an opinion leader. |
|  | **(Key Stakeholders) ^b^** | Stakeholder | 5 | Addressing different settings and work experience of dietitians, such as, students, freelancers and employees, those they just finished their education and those who have been in practice for many years. |

**^a^** Frequencies reflect the number of quotes on the topic and thus indicate how often the topic was discussed in the focus groups and not, consequently, the importance of the topic in general. **^b^** The construct key stakeholder is described in the CFIR codebook (63) however not mentioned as separate CFIR construct by Damschroder et al. (38).
